# Efficacy of Raman spectroscopy in the diagnosis of bladder cancer: A systematic review and meta-analysis

**DOI:** 10.1097/MD.0000000000018066

**Published:** 2019-11-22

**Authors:** Hongyu Jin, Tianhai Lin, Ping Han, Yijun Yao, Danxi Zheng, Jianqi Hao, Yiqing Hu, Rui Zeng

**Affiliations:** a Department of Liver Surgery, Liver Transplantation Center, West China Hospital; b West China School of Medicine; c Department of Urology, Institute of Urology, West China Hospital, Sichuan University, Chengdu, China; d Department of Cardiovascular Diseases, West China Hospital, Sichuan University, Chengdu, China.

**Keywords:** bladder cancer, Raman spectroscopy, sensitivity, specificity

## Abstract

**Background:**

Bladder cancer is one of the severest human malignancies which are hardly detected at an early stage. Raman spectroscopy is reported to maintain a high diagnostic accuracy, sensitivity and specificity in some tumors.

**Methods:**

We carried out a complete systematic review based on articles from PubMed/Medline, EMBASE, Web of Science, Ovid, Web of Knowledge, Cochrane Library and CNKI. We identified 2341 spectra with strict criteria in 9 individual studies between 2004 and 2018 in accordance to Preferred Reporting Items for Systematic Reviews and Meta-Analysis (PRISMA) guidelines. We summarized the test performance using random effects models.

**Results:**

General pooled diagnostic sensitivity and specificity of RS to kidney cancer were 94% (95% CI 0.93-0.95) and 92% (95% CI 0.90-0.93). The pooled positive LR was 10.00 (95%CI 5.66-17.65) while the negative LR was 0.09 (95%CI 0.06-0.14). The pooled DOR was 139.53 (95% CI 54.60-356.58). The AUC of SROC was 0.9717.

**Conclusion:**

Through this meta-analysis, we found a promisingly high sensitivity and specificity of RS in the diagnosis of suspected bladder masses and tumors. Other parameters like positive, negative LR, DOR, and AUC of the SROC curve all helped to illustrate the high efficacy of RS in bladder cancer diagnosis.

## 1 Introduction

Bladder cancer (BCa) is acknowledged as the fourth most common type of male genito-urinary tract malignancy and the eleventh female most common type of carcinoma, which accounts for more than 1% of the overall European population and also assumes 5% to 10% of cancers in the United States.^[1–3]^ Meanwhile, an approximate 75% to 85% of the diagnosed group suffer from non-muscle invasive type of BCa, in which a relatively high risk of repeated recurrence is predicted within 5 years following initial diagnosis.^[4,5]^ A generally accepted theory indicates that distinct biological pathways can induce BCa, which can be classified into low-risk individuals with corresponding low-grade papillary tumors unlikely to progress and high-risk individuals with carcinoma in situ (CIS) with high-grade tumors probable to develop.^[6]^ Therefore, techniques with promising efficiency of early diagnosis are pivotal in controlling tumor grades and stages.^[7]^

So far, a wide variety of diagnostic tests has been developed to efficiently detect early stage BCa, including computed tomography, magnetic resonance urography and intravenous urography, etc.^[8]^ However, these abovementioned diagnostic methods require the expertise of quite a large professional medical team, including radiologists, cytopathologists and clinical urologists.^[9]^ Thus, a delay caused by either section of the medical team can lead to an increased risk of cancer related death in the form of high-risk invasive BCa.^[10]^ Therefore, a crucial step to develop a non-expensive, non-invasive, rapid and automated diagnostic method with high sensitivity is mandatory.

Raman spectroscopy (RS) originates initially from analytical chemistry to evaluate chemical compounds based on varied excitation of vibrational modes in the internal chemical bonds.^[11]^ Since RS is able to detect Raman signals from the bonds within molecules, it can also provide the biochemical conditions within biological samples.^[12]^ Within a molecularly complicated biological system, like a cell, tissue or even an organ, RS carries intrinsic details and information of the materials present in the system, thus the biological status can also be acquired based on which the detailed features of the tissue are easily and accurately obtained.^[13,14]^

Because of the advantages of RS, an increased appliance of RS in the early and precise diagnosis of BCa has been reported.^[15]^ Meanwhile, a large number of clinical trials studying the possibility of using RS on epithelial cells from voided urines to detect early-stage malignancies have been carried out.^[16]^ However, results from different studies vary from each other. This can result from different numbers of people recruited, different nationalities of the people recruited or regional variations, etc.^[17]^ Thus, a comprehensive research to integrate the already published studies to acquire the most accurate and reliable data is proposed. This systematic review and meta-analysis aims to integrate these studies and come up with results depleted of potential errors and bias among them to provide the most reliable data concerning the efficiency of applying RS in the diagnosis of BCa.

## 2 Material and methods

### 2.1 Search strategy

We comprehensively and extensively searched several authenticated databases according to the guidelines for performing meta-analysis, including Web of Science, PubMed/Medline, Cochrane Library, China National Knowledge Infrastructure (CNKI) and ClinicalTrials.gov (http://www.ClinicalTrials.gov) for highly qualified and related articles published from January 2004 to December 2018. Articles found through the initial search were subsequently more strictly screened for their availability, quality and relevancy. No regional and language restriction were applied during the whole article searching and screening process.

### 2.2 Article selection

Two independent professional reviewers participated fully in the screening process to analyze the full texts to assess the quality, relevancy and availability to determine the ultimate recruited articles. We set primary inclusion criteria to firstly exclude studies without obvious values, these include:

1) reporting the use of RS in the diagnosis of BCa;
2) being a registered randomized controlled trials or applying all kinds of observational designs, including cross-sectional, case-control and cohort designs;
3) reporting at least the diagnostic sensitivity, specificity value, or other important parameters like true positive (TP), false positive (FP), true negative (TN) and false negative (FN) values, based on which sensitivity and specificity values could be calculated.

**Figure 1:**
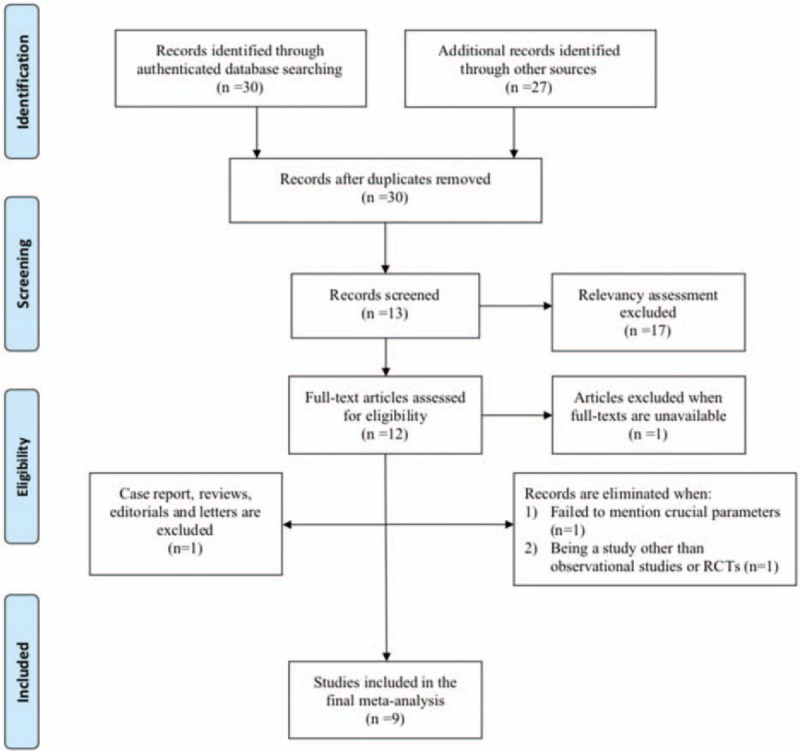
Flow-diagram of the article selection process.

Meanwhile, we particularly excluded studies which were letters, editorials, case reports, etc. The detailed exclusion criteria were shown in Figure 1.

During the process, we continuously performed a blinded cross-check to detect underlying discrepancies on any aspects. If a potential discrepancy was detected, we would assign a third professional investigator to consolidate the conflict in order to assure the accuracy of the data. The identification, inclusion and exclusion of articles were performed according to the PRISMA guidelines.

### 2.3 Data extraction

Two experienced investigators were subsequently assigned to extract important data after the articles were determined. Primary parameters indicating the diagnostic efficiency and basic information of the articles were extracted. Generally, 9 diagnostic efficiency related parameters including sensitivity, specificity, corresponding TP, TN, FP, FN values, accuracy and spectra data were extracted and further analyzed. Meanwhile, parameters concerning the basic characteristics of the studies, including article title, first author, publication year, nationality, department, ethnicity, study design, sex and median age of the patients, enrollment year were also carefully extracted.

### 2.4 Statistical analysis

Data were extracted on a study level when it was possible to construct a 2 × 2 table, based on which we calculated the sensitivity, specificity, positive predictive value (PPV), negative predictive value (NPV), odds ratio (OR) and diagnostic likelihood ratio (DLR) with their 95% CIs. The forest plots were generated in order to display sensitivity and specificity estimates through Meta-Disc version 1.4 (Clinical Biostatistics Unit, UK). To summarize test performance, 2 meta-analyzing diagnostic accuracy tests were used: the bivariate model and the hierarchical summary receiver operating characteristic (HSROC) model.^[18,19]^ We used these methods to respect the binomial structure of diagnostic accuracy data, thus jointly summarizing paired measures simultaneously, for example, sensitivity and specificity or, positive and negative LRs. Meanwhile, as a random effects approach, the bivariate/HSROC meta-analysis allowed pooling results in view of knowing that heterogeneity was commonplace across included studies due to different or implicit thresholds. The said approach was carried out by metandi (Meta-analysis of diagnostic accuracy using hierarchical logistic regression) command in STATA 14.2 (StataCorp, USA).

Meanwhile, summary receiver operator characteristics (SROC) curves were generated to determine the relationship between sensitivity and specificity. The area under curve (AUC) was simultaneously calculated to evaluate the overall performance. Theoretically, an excellent diagnostic effect was defined when AUC value was between 0.9 and 1; good when AUC value was between 0.8 and 0.9; fair when AUC value was between 0.7 and 0.8; poor when AUC value was between 0.6 and 0.7. The diagnostic method failed when AUC was between 0.5 and 0.6.^[20]^ The SROC curved was made through Meta-Disc version 1.4 (Clinical Biostatistics Unit, UK).

### 2.5 Quality assessment

Two independent reviewers simultaneously evaluated the methodological quality of each study via the QUADAS guidelines. All QUADAS items were applied to evaluate eligible studies. The articles were evaluated in the following processes: sequence generation (selection bias), allocation concealment (selection bias), blinding of participants and personnel (performance bias), blinding of outcome assessment (detection bias), incomplete outcome data (attrition bias), selective reporting (reporting bias) and others.

## 3 Results

### 3.1 Literature selection and screening

Initial literature research yielded 57 articles, including 30 records identified from authenticated databases and 27 studies found from other sources, in which only 30 studies were further considered after removing duplicates. Subsequently, 2 independent investigators performed relevancy evaluation and excluded another 17 studies. Within the 13 studies remained, full texts of 12 studies could be traced. We excluded articles failing to report crucial parameters or being a study other than any forms of observational studies or RCTs. Finally, we removed one study which was a case report. Eventually, 9 studies with reliable quality was considered for this meta-analysis. The process of study selection and screening process was shown in Figure 1.

### 3.2 Characteristics of the included studies

Among the 9 studies we included eventually, 6 studies were performed in Europe (1 in Scotland, 3 in the UK and 2 in Netherlands), 2 studies were carried out in China and 1 in the USA. The number of spectra collected varied from 40 to 1525, and wave length of spectra used were 785 nm in 7 studies, 632.8 nm in 1 study and 532 nm in 1 study. The included numbers of spectra were recorded in 8 studies, with a total number of 2341. Accuracy was recorded in 5 studies, ranging from 86% to 97%. The characteristics of the included studies were shown in Table 1.

**Table 1:**
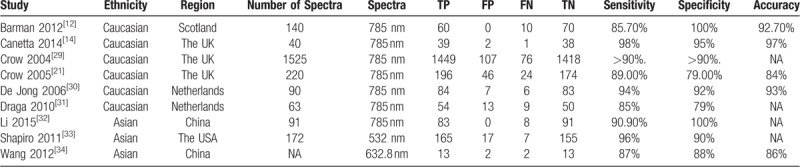
The characteristics of the included studies.

### 3.3 Pooled results

#### 3.3.1 Pooled sensitivity

**Figure 2:**
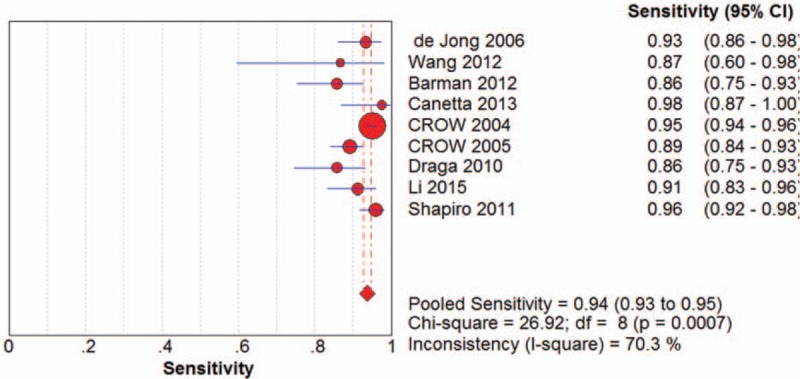
The forest plot of pooled sensitivity.

The sensitivity of the 9 included articles ranged from 86% (95% CI 0.75-0.93) in a study with 140 spectra to 98% (95% CI 0.87-1.00) which collected 40 spectra. The pooled sensitivity was 94% (95% CI 0.93-0.95), which indicated a comparatively low incidence rate of missed diagnosis. Particularly, among the 9 included studies, 5 studies maintained a sensitivity more than 90% and the additional 4 studies maintained a sensitivity between 85% and 90%. The forest plot of pooled sensitivity of all the 9 studies was shown in Figure 2.

#### 3.3.2 Pooled specificity

**Figure 3:**
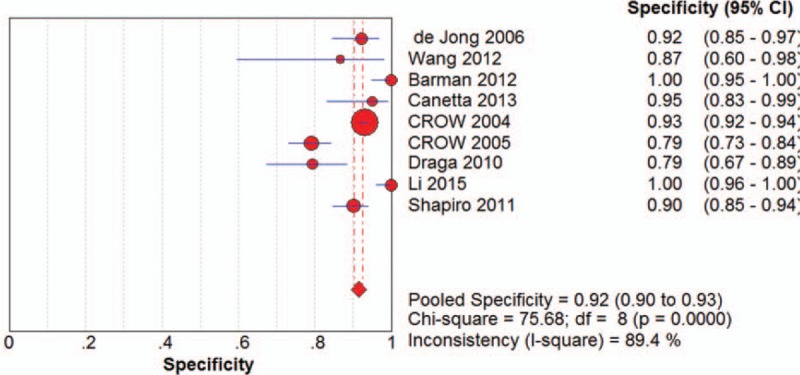
The forest plot of pooled specificity.

The specificity of the 9 included studies ranged from 79% (95% CI 0.67-0.89) in studies with 63 spectra and 220 spectra respectively to 100% (95% CI 0.95-1.00) and 100% (95% CI 0.96-1.00) by studies with 140 spectra and 91 spectra respectively. The general pooled specificity was 92% (95% CI 0.90-0.93), which was also a satisfactory parameter indicating a comparatively low rate of incorrect diagnosis. The forest plot of pooled specificity of all the seven studies was shown in Figure 3.

#### 3.3.3 Other parameters

**Figure 4:**
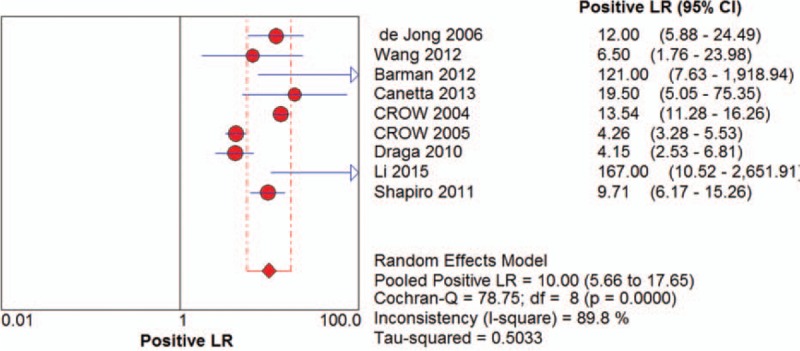
The forest plot of pooled positive LRs.

**Figure 5:**
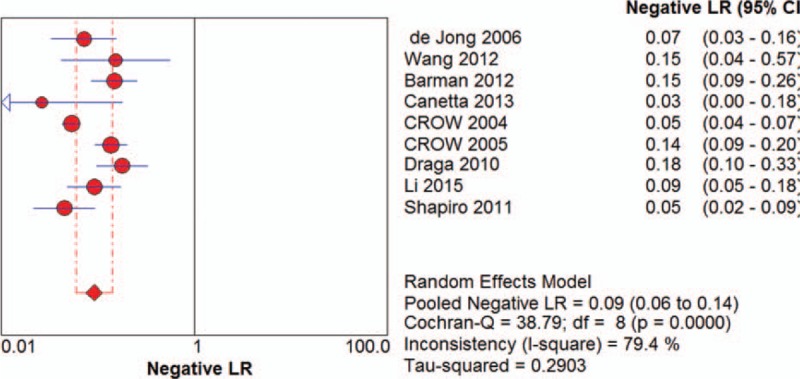
The forest plot of pooled negative LRs.

**Figure 6:**
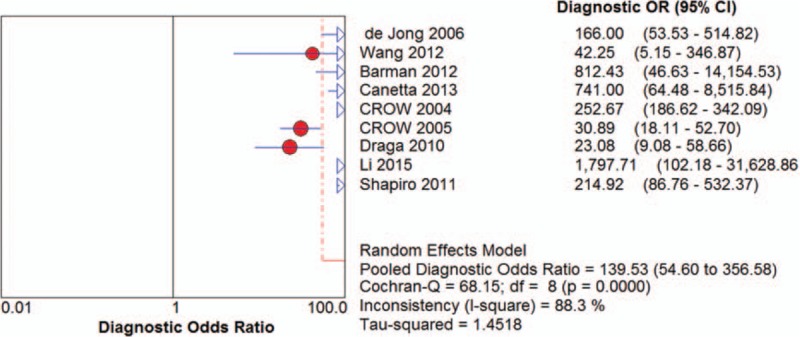
The forest plot of pooled DORs.

**Figure 7:**
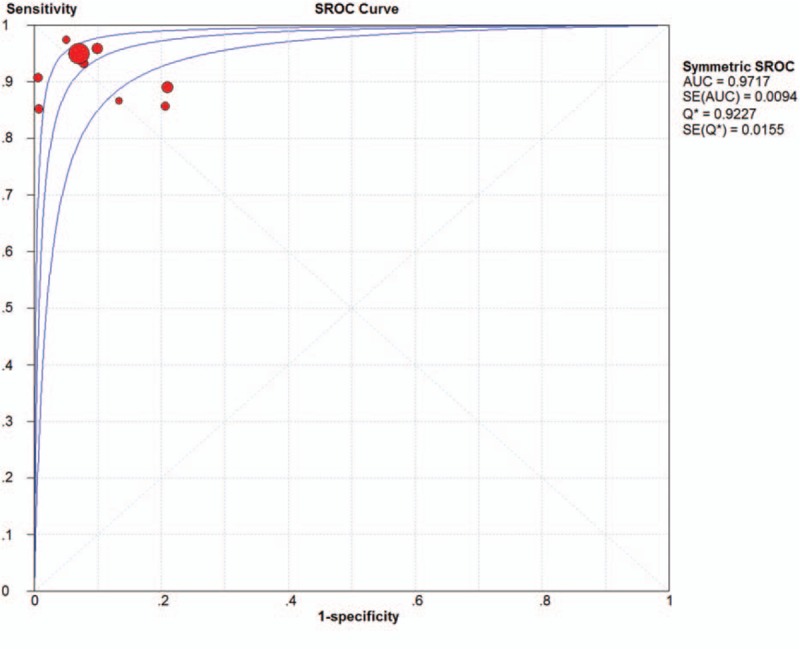
The SROC curve.

Among the recruited studies, positive likelihood ratios (LRs) ranged from 4.15 (95% CI 2.53-6.81) to 167.00 (95% CI 10.52-2651.91) with a pooled positive LR of 10.00 (95%CI 5.66-17.65) by random effects model. The forest plot of positive LRs was shown in Figure 4. Of all the studies, negative LR ranged between 0.03 (95% CI 0.00-0.18) and 0.18 (95% CI 0.10-0.33) with the pooled negative LR of 0.09 (95%CI 0.06-0.14) by random effect model. The forest plot of negative LRs was shown in Figure 5. Based on positive and negative LRs, we pooled the diagnostic odds ratios (DORs) of the included studies and found the DORs ranged between 23.08 (95%CI 9.08-58.66) and 1797.71 (95%CI 102.18-31628.86) with the pooled DOR of 139.53 (95% CI 54.60-356.58) by random effect model. The forest plot of DOR was shown in Figure 6. The AUC was 0.9717. The SROC curve was displayed in Figure 7.

## 4 Discussion

This systematic review and meta-analysis was carried out in accordance with the standard protocol for systematic reviews, recruiting a total of 9 articles and more than 2341 spectra into consideration. Two independent reviewers were assigned in study screening, quality assessing, data collecting and analyzing process. When necessary, a third reviewer was assigned to process the divergence from the first two reviewers. Methodologies including heterogeneity exploration and SROC curve analysis were simultaneously applied.

This systematic review and meta-analysis re-confirmed the superiority and high diagnostic efficacy of RS in the diagnosis of BCa, in accord with several previous clinical trials. Through this meta-analysis, we calculated the general pooled diagnostic sensitivity and specificity of RS to BCa, which were 0.94 (95% CI 0.93-0.95) and 92% (95% CI 0.90-0.93). In a word, a sensitivity and specificity over 90% were observed which illustrated a high efficacy of early diagnosis of suspected bladder lesion and mass. These data also stated a comparatively low incidence of both missed and incorrect diagnosis. The diagnostic accuracy was recorded in 5 studies ranging from 86% to 97%, which was also quite satisfactory. Tradition diagnostic methods like imaging, ultrasonography and even more recent one as fluorescence in situ hybridization (FISH) only manifest ordinary clinical diagnostic efficiencies, reflected by diagnostic sensitivity, specificity and parameters alike.^[21]^ Thus, Adding more detailed explanation and references compared with traditional diagnostic methods with a relatively fluctuating diagnostic efficacy, RS provided more stable and accurate data. Moreover, we found a general pooled DOR of 139.53 (95% CI 54.60-356.58) by random effect model, with the smallest DOR in a single study of 23.08 (95%CI 9.08-58.66). Since a DOR over 1 indicates a high discriminant effect and discriminant effect increases with DOR value, the general DOR of RS in diagnosing BCa accounted for a trustworthy diagnostic efficacy. In SROC curve analysis, AUC was 0.9717. According to the standard grading system for SROC, the diagnostic efficiency was regarded as excellent.

Crow et al investigated the efficacy of RS on bladder tissue by collecting different measured spectra from both malignant and benign tissues.^[22]^ Their team demonstrated a comparatively high efficacy of RS in distinguishing between normal and harmful tissues. Shapiro et al used a 532 nm excitation light in place of traditional 785 nm. Their experiment found this wavelength provided a more useful Raman spectrum with higher Raman scattering relative to excitation wavelengths in more red spectral regions. Besides noninvasiveness and accuracy, RS also maintains quite a number of advantages compared with traditional diagnostic methods. First, RS is fast, which takes only 15-50 min depending on the cleanliness of the urine. Second, it can be carried out without the help from spectroscopists and pathologists. Moreover, RS will not produce unnecessary waste. Therefore, future studies are expected to focus on identifying the most suitable wavelength to increase the diagnostic efficacy to a greater extent.

Shariat et al proposed in a prospective study that some BCa patients might have been excessively diagnosed.^[23]^ His study found that an approximate 40% of 900 BCa patients were excessively diagnosed by pathological analysis, while 20% of BCa patients were underestimated. However, RS combined with PCA/SVM diagnosis can lower the possibility of both excessive diagnosis and underestimation, which is preferable for the purpose of early and accurate diagnosis.

However, besides the use of RS, other parameters concerning the diagnostic efficacy and prognostic prevention should also be paid great attention. In 2015, Ferro et al found modified Glasgow prognostic score was associated with risk of recurrence in bladder cancer after radical cystectomy.^[24]^ In 2017, Busetto et al found the amount of circulating tumor cells (CTCs) was related to the prognosis of non-muscle-invasive bladder cancer.^[25]^ In the same year, a research ream found the urinary long non-coding RNAs had prognostic value in non-muscle invasive bladder cancer.^[26]^ In 2018, some researchers found that neutrophil to lymphocyte ratio was a strong prognostic predictor in patients with primary T1 HG/G3 non-muscle-invasive bladder cancer.^[27]^ Another group of scientists found systematic inflammatory biomarkers are related to the oncological outcomes in patients with high-risk non-muscle-invasive urothelial bladder cancer.^[28]^

Still, we acknowledged several limitations in this study. First, RS has not been widely accepted as a conventional clinical diagnostic tool, therefore inadequate number of clinical researches was published, which to some extent lowered the number of articles we could recruit. Second, the standard process and protocol of RS diagnosis have not been built, therefore it's hard to standardize the process of RS.

## 5 Conclusion

Through this meta-analysis, we found a promisingly high sensitivity and specificity of RS in the diagnosis of suspected bladder mass and tumors. Other crucial parameters including positive, negative LR, DOR, and AUC of the SROC curve all contributed to illustrate the preferable efficacy of RS in BCa diagnosis.

## Author contributions

**Conceptualization:** Hongyu Jin, Tianhai Lin, Ping Han, Rui Zeng.

**Data curation:** Hongyu Jin, Danxi Zheng.

**Formal analysis:** Hongyu Jin, Tianhai Lin, Yijun Yao, Yiqing Hu.

**Funding acquisition:** Tianhai Lin, Rui Zeng.

**Investigation:** Hongyu Jin, Tianhai Lin, Yijun Yao, Jianqi Hao.

**Methodology:** Hongyu Jin, Yijun Yao, Jianqi Hao.

**Project administration:** Rui Zeng.

**Resources:** Yijun Yao.

**Software:** Hongyu Jin.

**Supervision:** Ping Han, Rui Zeng.

**Validation:** Tianhai Lin, Ping Han, Rui Zeng.

**Visualization:** Tianhai Lin, Ping Han, Rui Zeng.

**Writing – original draft:** Hongyu Jin, Tianhai Lin, Rui Zeng.

**Writing – review & editing:** Hongyu Jin, Ping Han, Rui Zeng.
